# SIDECACHE: Information access, management and dissemination framework for web services

**DOI:** 10.1186/1756-0500-4-182

**Published:** 2011-06-14

**Authors:** Mark S Doderer, Cory Burkhardt, Kay A Robbins

**Affiliations:** 1 Greehey Children's Cancer Research Institute, The University of Texas Health Science Center at San Antonio, San Antonio, TX 78229, USA; 2 eMetric LLC, San Antonio, TX 78232, USA; 3 Department of Computer Science, The University of Texas at San Antonio, San Antonio, TX 78249, USA

## Abstract

**Background:**

Many bioinformatics algorithms and data sets are deployed using web services so that the results can be explored via the Internet and easily integrated into other tools and services. These services often include data from other sites that is accessed either dynamically or through file downloads. Developers of these services face several problems because of the dynamic nature of the information from the upstream services. Many publicly available repositories of bioinformatics data frequently update their information. When such an update occurs, the developers of the downstream service may also need to update. For file downloads, this process is typically performed manually followed by web service restart. Requests for information obtained by dynamic access of upstream sources is sometimes subject to rate restrictions.

**Findings:**

SideCache provides a framework for deploying web services that integrate information extracted from other databases and from web sources that are periodically updated. This situation occurs frequently in biotechnology where new information is being continuously generated and the latest information is important. SideCache provides several types of services including proxy access and rate control, local caching, and automatic web service updating.

**Conclusions:**

We have used the SideCache framework to automate the deployment and updating of a number of bioinformatics web services and tools that extract information from remote primary sources such as NCBI, NCIBI, and Ensembl. The SideCache framework also has been used to share research results through the use of a SideCache derived web service.

## Background

Often bioinformatics researchers deploy new methods as web services to make them easily accessible in client browser applications or from other tools. For example, BioCatalogue [1] currently curates more than 1,700 life science web services and the number is rapidly growing. A typical bioinformatics web service performs a calculation or directly returns pre-computed information based on a user request. Many such services rely on or include information consolidated from other sites.

A difficulty with this distribution strategy is that many major sources of bioinformatics information such as NCBI are regularly updated. Developers are then faced with the task of periodically re-downloading the data and rerunning computations in order to keep their results up-to-date. Users may find that the results based on the new information are not the same as the results obtained from earlier requests, but usually they have no way of knowing what information was used nor do they have the option of rolling back to a previous data state.

The origin of data or provenance has received much attention in both the database community and in the scientific workflow community [2,3]. Detailed provenance information can be represented by directed acyclic graphs and exchanged using the Open Provenance Model (OPM) [4]. The Taverna Workbench [5] is an example of a workflow system that allows the development of web services based on pipelines or workflows. Developers can create new web services by constructing workflows of available components. Taverna incorporates an internal data model called Janus and supports provenance queries on the workflows. The CaGrid Workflow Toolkit [6] is an example of a Taverna extension that focuses specifically on the CancerBiomedical Informatics Grid (CaBig) and supports provenance queries.

Database and workflow systems implement "fine-grained" provenance models, which trace origin of individual pieces of data and individual components in a calculation. Unfortunately, most public web sources do not currently make fine-grained provenance information available to their clients. Furthermore, most client side tools do not yet support facilities for making such information usable. Chapman and Jagadish [7] have examined the issue of usability in the provenance information available in workflow systems and have shown the mismatch between the types of provenance questions users are likely to ask and the types of information stored to establish the provenance of a workflow. Very few web services provide provenance information of any kind.

SideCache is a simple deployment framework that allows developers to specify a schedule for automatically updating a web service and includes a version number with each user query. Users can examine available versions and obtain results based on previous versions as well as on the current version. Developers can include information about the implementation and data sources as metadata, which is captured with each version and returned upon user query. While not a substitute for true provenance, the SideCache approach allows developers to quickly deploy data web services without having to return in subsequent months and years to run updates.

SideCache also provides caching and rate control for requests to external sites. For security reasons, much of the client side browser technology can only issue web service requests to servers in the domain from which a page was downloaded unless the remote site provides certain certificates (which most do not). Thus, applications making client-side web requests are generally forwarded through their originating server. However, many bioinformatics sites (such as NCBI) have strict restrictions on the rate at which a particular IP address can make web service requests. While the requests of an individual client may not exceed these rates, the situation can easily become serious when requests from multiple clients are pooled. NCBI states that if queries from an IP address exceed a set rate per second, the IP address will be flagged and future queries will go unfulfilled until the address is removed from the NCBI banned IP list. The SideCache Proxy web services address these issues by providing local caching and pooled rate restriction for remote sites as specified by the deployer. These facilities are important for deployment of services that require some on-the-fly queries to external sites.

### Implementation

**Figure 1:**
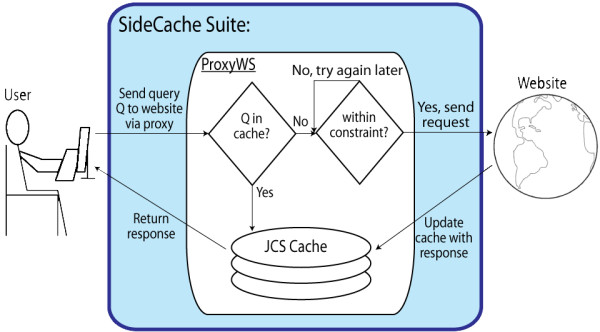
**User sends a query to an external website via ProxyWS**.

The SideCache ProxyWS shown in Figure 1 provides caching and rate control. A user client accesses information by making a query Q to a remote website using a server running the ProxyWS web service as an intermediary. This user might request remote information using a browser with an XHTML form and JavaScript, some other client-side browser application, or a standalone desktop application. If the request matches locally stored information, ProxyWS returns that information immediately without issuing a request to the remote server. Otherwise, ProxyWS issues the remote request and caches (saves) the response in addition to returning it to the client.

If the request represented by Q in Figure 1 does not match locally stored information, ProxyWS checks to see if the call will violate the access rules and time constraints specified by the deployer and only makes the remote call if the request satisfies the constraints. Otherwise, ProxyWS returns a failure, expecting its client to retry the request. ProxyWS saves remote responses in its JCS [8] cache in order to satisfy future requests.

**Figure 2:**
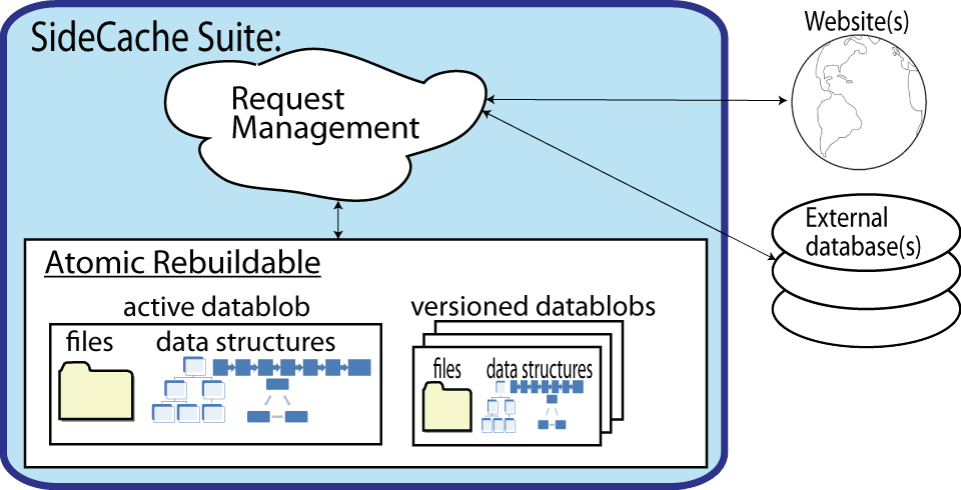
**The atomic rebuildable data blob**.

Web services that require file downloads and recomputation are implemented in SideCache by extending a rebuildable service class. Each SideCache rebuildable data web service satisfies its requests from a read-only atomic rebuildable data blob as illustrated in Figure 2. The data blob may include files, data bases, and internal data structures. The data blob is identified by a version number and stored in a subdirectory identified by that version. The data blob also includes the metadata contained in the configuration file that was in effect at the time of blob creation.

**Figure 3:**
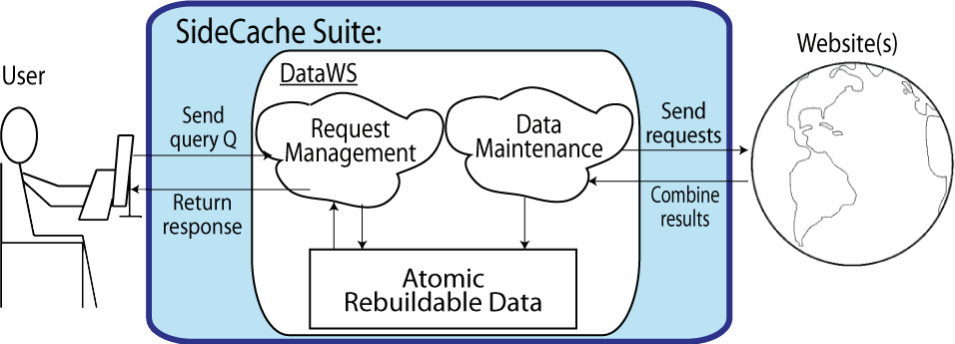
**User sends a query to a DataWS**.

Figure 3 shows a schematic of the SideCache update architecture. SideCache handles data maintenance independently of request management using the update schedule provided in the developer-specified configuration. SideCache attempts to create new versions according to the update schedule by performing necessary file downloads and other types of accesses and running the processing programs needed to generate a new blob. If this process succeeds in its entirety, SideCache uses synchronization to make the new version the current version, while retaining older versions (according to schedule). Clients in the process of making a web service request while this process is proceeding operate on the previous version without disturbance.

### Deployment

The SideCache Suite is implemented in Java and designed for services running as Java servlets under a servlet container such as Apache Tomcat [9]. The SideCache infrastructure is available as a Java .jar file that can simply be placed in the Tomcat library directory. The data and proxy web services are deployed using Tomcat's manager. To implement and deploy a rebuildable data web service using SideCache, a developer defines the external data sources and specifies a download schedule in a configuration file (See Additional File 1: Deployment.pdf for details.) The developer then extends SideCache's RebuildableService class by providing a performOperation method that responds to the web service request and a createRebuildableData method that creates the needed data structures and files for the data blob that supports the service.

To deploy a proxy web service, the developer specifies the access information and rate constraints in an XML file. Additional File 1 provides an example of such a service deployment.

## Results and Discussion

We use the SideCache suite in a number of our own web services and as the basis for the Sidekick [10] client side application. Table 1 summarizes three services currently running under our own SideCache deployment at visual.cs.utsa.edu. The GeneInfoWS provides basic information about genes from NCBI based on NCBI gene IDs. This service also provides a look up of NCBI gene ID from gene name for selected species. Our implementation downloads gene information files from NCBI weekly and creates in memory look-up tables. Interaction information is stored in files.

**Table 1:** Example services using various SideCache features

Web Service / Type / Operation	Purpose/Example	Data Source
GeneInfoWS data web service getInfo	Inputs a list of NCBI gene IDs and returns name, chromosome number, and chromosome start position of the corresponding genes. http://visual.cs.utsa.edu/GeneInfoWS?operation=getInfo&sourceSpecies=9606&genes=7157,20,672	NCBI

GeneInfoWS data web service getIds	Inputs a list of gene names and returns a list of the corresponding NCBI gene IDs. http://visual.cs.utsa.edu/GeneInfoWS?operation=getIds&sourceSpecies=9606&genes=TP53,ABCA2,BRCA1	NCBI

GeneInfoWS data web service getInteractants	Inputs a list of NCBI gene IDs and returns a list of NCBI gene IDs for the genes whose products interact with the products of the input genes. The results for both input and output gene IDs include gene name, chromosome and chromosome start position. http://visual.cs.utsa.edu/GeneInfoWS?operation=getInteractants&sourceSpecies=9606&genes=7157,20,672	NCBI

GeneInfoWS data web service getVersions	Returns a list of provenance data including a version identifier as well as creation date, size and source for each file. http://visual.cs.utsa.edu/GeneInfoWS?operation=getVersions	

GeneInfoWS data web service versioned getInfo	Inputs a list of NCBI gene IDs and returns name, chromosome number, and chromosome start position of the corresponding genes. http://visual.cs.utsa.edu/GeneInfoWS?operation=getInfo&sourceSpecies=9606&genes=7157,20,672&versionIdentifier=1296771389679(getid)	NCBI

EnrichWS data web service goEnrichment	Inputs a list of NCBI gene IDs, GO type, IEA evidence inclusion, a maximum group count and species. The service returns groups of gene IDs that are enriched for particular GO terms. The results include term, group members and group p-value. The number of groups is limited by the input group count. http://visual.cs.utsa.edu/EnrichWS?enrichType=goEnrichment&calc=Parent-Child-Union&species=9606&genes=1499|7827|2719|5002&max=10&evid=_IEA_&type=Component	Gene ontology from NCBI

EnrichWS data web service meshEnrichment	Inputs a list of NCBI gene IDs, a maximum group count, and taxonomy ID. The service returns the enriched MeSH terms, their corresponding enriched members, and the p-value for that group. The number of groups is limited by the input group count. http://visual.cs.utsa.edu/EnrichWS?enrichType=meshEnrichment&calc=Parent-Child-Union&species=9606&genes=1499|7827|2719|5002&max=10	Gene2MeSH from NCIBI

EnrichWS data web service getVersions	Returns a list of provenance data including a version identifier as well as creation date, size and source for each file. http://visual.cs.utsa.edu/EnrichWS?enrichType=getVersions	

OrthologWS data web service default	Inputs a list of NCBI gene IDs, the source species taxonomy ID, and the target species taxonomy ID. The service returns all orthologs and the percent identity shared between the products of the source and target genes. http://visual.cs.utsa.edu/OrthologWS?sourceSpecies=9606&targetSpecies=10090&genes=7157,20,672	Ensembl

OrthologWS data web service getVersions	Returns a list of provenance data including a version identifier as well as creation date, size and source for each file. http://visual.cs.utsa.edu/OrthologWS?operation=getVersions	

ProxyWS proxy web service uml	Returns the results from the input UML. Manages caching for efficient retrievals, and timing of external web site calls. http://visual.cs.utsa.edu/ProxyWS/Proxy?url=http://eutils.ncbi.nlm.nih.gov/entrez/eutils/esummary.fcgi?db=gene&id=7157,20,672&retmode=xml	

The EnrichWS performs enrichment analysis for an input list of NCBI gene IDs using the enrichment approach of Grossman et al. [11]. GO enrichment is calculated from downloaded NCBI gene ontology files. The mesh enrichment service is derived from NCIBI's Gene2MeSH [12], which integrates public sources of information to associate genes with Medical Subject Headings (MeSH terms) [13]. Gene2MeSH does not provide download files for its information, so calls must be done dynamically. We configured these services to perform the calls through ProxyWS in order to cache the information locally and avoid network calls to remote sources. If the web service is restarted, SideCache ProxyWS will service the dynamic calls using local information. Finally, the OrthologWS periodically queries Ensembl's Biomart [14] web service, downloads identifier and orthology information, and creates indexed files. The OrthologWS returns the orthologous genes from look-ups using these indexed files.

To test the efficacy of ProxyWS, we repeatedly restarted a simple data web service after deleting the working files. The forced recovery necessitated a download of files from the external web sources. As summarized in Table 2, the time for recovery proved on average to be roughly 400% faster for the cached downloads than for non cached downloads.

**Table 2:** Time comparison for cached and non-cached downloads

URL of source	Size of File (MB)	Times for Cached Calls (MS)	Times for Non-Cached Calls (MS)
ftp://ftp.ncbi.nlm.nih.gov/gene/DATA/GENE_INFO/Fungi/Saccharomyces_cerevisiae.gene_info.gz	1.5	Avg. 529 Min 76 Max 2263	Avg. 2799 Min 2022 Max 4002

ftp://ftp.ncbi.nlm.nih.gov/gene/DATA/GENE_INFO/Invertebrates/Caenorhabditis_elegans.gene_info.gz	2.7	Avg. 637.4 Min 135 Max 2612	Avg. 2438.8 Min 1820 Max 3170

ftp://ftp.ncbi.nlm.nih.gov/gene/DATA/GENE_INFO/Invertebrates/Drosophila_melanogaster.gene_info.gz	3.8	Avg. 1145.6 Min 193 Max 4944	Avg. 3496.6 Min 2601 Max 4134

ftp://ftp.ncbi.nlm.nih.gov/gene/DATA/GENE_INFO/Mammalia/Homo_sapiens.gene_info.gz	9.4	Avg. 2003 Min 493 Max 7917	Avg. 12088 Min 5998 Max 32284

ftp://ftp.ncbi.nlm.nih.gov/gene/DATA/GENE_INFO/Mammalia/Mus_musculus.gene_info.gz	11.2	Avg. 2076.2 Min 681 Max 7344	Avg. 9371.2 Min 7751 Max 12110

**Totals**	**28.6 MB**	**1278.2 MS**	**6038.7 MS**

Many algorithms and methods in bioinformatics are based on publically available date that is continuously updated. An automatic update system such as SideCache allows developers to deploy algorithms as web services and to specify the frequency of updates of the underlying data without further manual intervention. The SideCache versioning system includes a version with each data rebuild. The versioning allows users to retrieve information about the available versions and to address a query to a previous version. SideCache user responses automatically include information about what external resources were downloaded and the date of download as well as provenance annotations provided by the deployer. While not a fine-grained provenance system, versioning provides enough information for users to recreate earlier responses and to back trace information from external sources. The frequency of updates is left to the developer's discretion. For example, although NCBI updates its data daily, the web service described in this paper is only updated weekly because the service does not warrant the storage cost for more frequent updates. Many journals require authors to explicitly state the update frequency of the services described in their publications. SideCache allows web service creators to automate these updates.

If a scheduled update fails, SideCache logs the errors. SideCache only replaces the current data blob if all of the external data has been downloaded and the computation completed successfully. Occasionally external web services will change the calling URL or format of the results, which will require modifications to the download settings and processing methods.

SideCache enables the developer of a web service to take a data warehousing approach to versioning by saving each version as an immutable data blob. Deployers specify how long to retain previous versions or may specify that the versions be kept indefinitely. The deployment strategy of saving copies of previous versions may not work well for services involving very large data sets. However, the availability of multi-terabyte drives makes this strategy feasible for most services. This strategy fits biological research well. When new information is discovered it is included in the developer's data repositories, however previous data corresponding to previous publications must be retained. The developer simply indicates the published version data identifier and even when new versions are added with each update the previous data will be available.

Using web services to distribute data and algorithms increases their likelihood of use by providing easy programmatic as well as viewing access. The example services of this paper return information in a variety of formats including html, xml, csv, text files, and zipped collection of files. Formats such as html can be displayed in the requestor's browser, while xml and downloadable zip files allow the integration with other services. Many public data repositories such as FlyBase [15] and Mouse Genome Database [16] enable links out from their web sites, if the linked website provides the format of its URL and a list of synonyms for source website identifiers on the linked website. These requirements are easy to manage with a data web service.

As is typical of caching systems, ProxyWS avoids the cost of remote communication at the price of local storage and access. The developer specifies in a configuration file which sites should be cached. Good candidates for caching require multiple remote web service requests rather than a single file download. The ProxyWS can also provide two-level caching for users in a lab or those sharing an intranet. By deploying a commonly-used service through the proxy running on a local server, users with common interests can share downloaded information, improving efficiency and reducing external internet accesses.

An example of the value of SideCache comes from our own experience with InterologFinder [17], a web application that was originally published prior to the implementation of the SideCache framework. InterologFinder was based on complex HTML display of results that had been computed at the time of publication. To update this information, the authors had to rerun all of the calculations produced for the paper. Recently, the SideCache framework was used to re-implement and enhance InterologFinder. InterologFinder now automatically re-computes its results as new information becomes available and also exposes its results as a web service.

## Conclusions

SideCache provides a framework that enables a research organization to manage data more efficiently including gathering and parsing external data and sharing newly published research results. The proxy web service manages timing calls for data sources with developer-created access policies and implements a caching system to optimize web service requests. SideCache also provides a simple framework for deploying data web services by providing added functionality in the form of automatic downloading and parsing of external data combined with the ability to develop in-house data analysis tools that are simple to share through the group's local web server. SideCache similarly makes it relatively straightforward to develop and deploy mechanisms to share research results. The data web services include a simple method for handling coarse grain provenance as the information evolves over time.

## Availability and requirements

• **Project name: **SideCache

• **Project home page: **http://visual.cs.utsa.edu/sidecache.html

• **Operating system(s): **Platform-independent with a servlet container (for example Apache Tomcat)

• **Programming language: **Java

• **License: **No license required

• **Any restrictions to use by non-academics: **None

## Competing interests

The authors declare that they have no competing interests.

## Authors' contributions

MSD, CB and KAR all participated in the development of the framework. MSD and KAR prepared the manuscript. All authors read and approved the final manuscript.

## Supplementary Material

Additional File 1 **Deployment.pdf **A detailed description of the steps to create and deploy a proxy and data web service. Click here for file
