# Supplementary material for: SIDECACHE: Information access, management and dissemination framework for web services
Source: BMC Res Notes. 2011 Jun 14;4:182. doi: 10.1186/1756-0500-4-182 (PMC3132714; doi:10.1186/1756-0500-4-182)
Supplement: Additional File 1 — Deployment.pdf A detailed description of the steps to create and deploy a proxy and data web service. [file 1756-0500-4-182-S1.PDF]

Supplemental Material - Deployment.pdf

This supplement describes the process that a developer goes through to deploy a SideCache proxy web service and a SideCache rebuildable data web service. The services are implemented using servlets and deployed using Apache Tomcat as the servlet container. The jar and zip files referred to in the discussion are available on `http://visual.cs.utsa.edu/sidecache.html`.

S.1 SideCache Proxy web service

Overview: SideCache's core functionality of caching and management of timed calls to external data sources is implemented by `ProxyWS`, which extends the Java `RebuildableService` class. `ProxyWS` takes an input URL and returns previously cached results. If `ProxyWS` does not find the URL among previously cached results, it checks to make sure that a query to the specified source would fall within the specified rate restrictions. If the query falls within frequency restrictions, `ProxyWS` sends the query to the target web source, adds the result to the cache, and returns the result to the original requestor. If the query falls outside the frequency requirements, `ProxyWS` sends a response error with the expectation that the user of the proxy will retry the request. To deploy `ProxyWS` for a specific application, a developer must do three things:

1. Specify the remote sites and their access restrictions
2. Specify the external name by which the web service will be known
3. Deploy the servlet in a servlet container (Tomcat for our examples)

The `ProxyWS.zip` file contains all of the files (seen in Figure S1) needed for proxy web service deployment to an Apache Tomcat server.

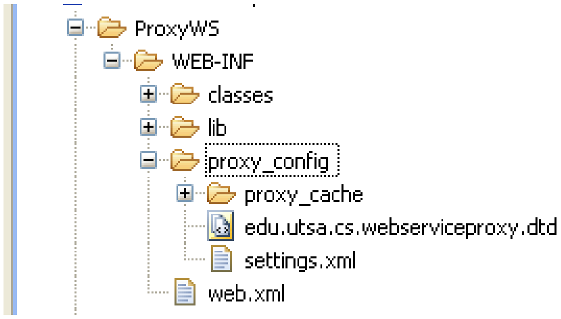

Figure S1 – Directory structure of a typical proxy web service

1. Specify the remote sites and their access restrictions: The developer specifies the sites to be accessed through the proxy web service in a file called `settings.xml` as shown in Figure S2.

```
<Proxies>
    <ProxyConfig>
       <Cache type="indexed">
          <CacheName>WebServiceProxy</CacheName>
          <CacheLocation>proxy_cache</CacheLocation>
          <MemoryCacheSize>10</MemoryCacheSize>
          <DiskCacheSize>50000</DiskCacheSize>
       </Cache>
       <Service>
          <HostName>http://eutils.ncbi.nlm.nih.gov</HostName>
          <HostPort></HostPort>
          <HostService></HostService>
          <ServiceMethod>Post</ServiceMethod>
          <Rules>
             <MaxRequestRate numberRequests="180" secondsDuration="60" />
             <ExpirationHours>72.0</ExpirationHours>
          </Rules>
       </Service>
       <Service>
          <HostName>http://www.pathwaycommons.org</HostName>
          <HostPort></HostPort>
          <HostService></HostService>
          <ServiceMethod>Get</ServiceMethod>
          <Rules>
             <MaxRequestRate numberRequests="0" secondsDuration="0" />
             <ExpirationHours>-1</ExpirationHours>
          </Rules>
       </Service>
    </ProxyConfig>
</Proxies>
```

Figure S2 – settings.xml example

The settings.xml file has a <Service> specification for each external site that includes the target site access information, rate control information, and cache expiration information. The cache specified by Figure S2 is configured to hold 10 objects in memory (<MemoryCacheSize>) and 50,000 disk objects (<DiskCacheSize>). A developer can eliminate one of these cache storage entities by assigning its corresponding size specification to 0. To remove caching and use the proxy web service for rate control only, the developer simply sets both size elements to 0. This configuration supports target sites located at http://eutils.ncbi.nlm.nih.gov and at http://www.pathwaycommons.org. The first example limits target access to 180 requests in 60 seconds. Cache entries expire after 72 hours. Since the <HostPort> and <HostService> are not specified, SideCache assumes the specification applies to the default port 80 and all services associated with this host. The second example has a similar access values, however the cache entries for this site do not expire (although they may be evicted based on space considerations) due to the specification <ExpirationHours>-1</ExpirationHours>. The <ServiceMethod> specifies the communication method for the web service. If a host provides Post access for a specific service and Get access for another, the developer will use <HostService> to explicitly set each.

The edu.utsa.cs.webserviceproxy.dtd file describes the allowed syntax for the settings.xml. The settings.xml and DTD files together with the Java class files in the classes directory specify the core functionality of ProxyWS.

2. Specify the external name by which the web service will be known: The Tomcat server parses the web.xml file when it activates the servlet. Figure S3 shows an example of this file. In this example, the servlet and main directory are both named ProxyWS (<servlet-name>) for

readability, although this is not needed. The `<servlet-mapping>` element specifies how this servlet is known to the outside world.

```
<?xml version="1.0" encoding="UTF-8"?>
<web-app id="WebApp_ID" version="2.4"
xmlns="http://java.sun.com/xml/ns/j2ee"
xmlns:xsi="http://www.w3.org/2001/XMLSchema-instance"
xsi:schemaLocation="http://java.sun.com/xml/ns/j2ee
http://java.sun.com/xml/ns/j2ee/web-app_2_4.xsd">

    <servlet>
       <servlet-name>ProxyWS</servlet-name>
       <servlet-class>
          edu.utsa.servlet.WebServiceProxyServlet
       </servlet-class>
       <load-on-startup>1</load-on-startup>
    </servlet>

    <servlet-mapping>
       <servlet-name>ProxyWS</servlet-name>
       <url-pattern>/Proxy</url-pattern>
    </servlet-mapping>
</web-app>
```

Figure S3– `web.xml` example

The URL call to this web service includes `/Proxy` (`<url-pattern>`) and specifies the required path for users of the web service. All of the other settings, including the path to the main class `edu.utsa.servlet.WebServiceProxyServlet` (`<servlet-class>`), should not be changed for a new proxy web service. The URL for this example (which retrieves results from NCBI through the proxy web service) is:

`http://host.edu/ProxyWS/proxy?url=http://eutils.ncbi.nlm.nih.gov`

3. Deploy servlet in the container: After making the changes, the developer must put the files in the correct place for Apache Tomcat. Several methods are available for deployment using Tomcat. We describe two of the simplest, both of which require the file structure within the zip file to be combined into a single `.war` file. A WAR file (web application archive) is a jar file used to distribute java-based web applications. Some archive programs export jar files. A user

with Java installed can also use the `jar –cvf` on the command line. For this example, if the developer is in the `ProxyWS` directory shown in Figure S1 and `jar` is in the class path for the system, the command `jar –cvf ProxyWS.war *` archives the `WEB-INF` directory and all of its subdirectories into a file named `ProxyWS.war`. The Tomcat manager, if installed, accepts this file for deployment using the "WAR file to deploy " option. Alternatively, if Tomcat's `autoDeploy` setting is true (see Tomcat's `server.xml` file), the developer simply copies the WAR file into the Tomcat's application base directory (`webapps` by default), and Tomcat automatically starts the servlet.

S.2 SideCache Rebuildable Data web service

To build and deploy a rebuildable data web service, use the supplied `GenericWS.zip` file as a template. Import `GenericWS.zip` into a Java program development environment such as Eclipse, creating the file structure shown in Figure S4.

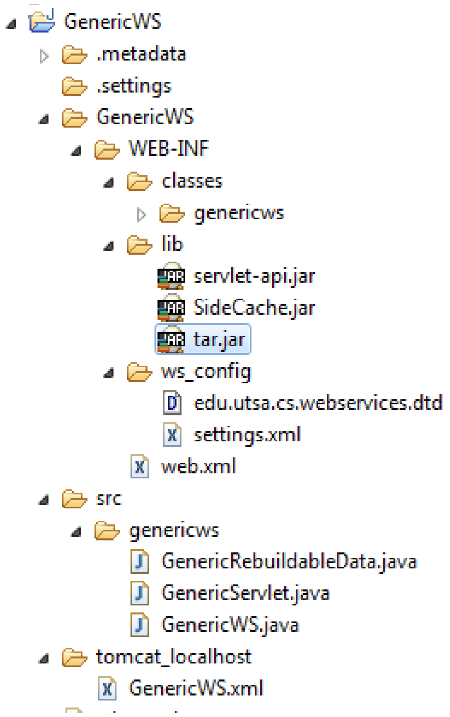

Figure S4 - File structure of the template data web service

Do the following steps, which are described in more detail below:

1. Specify the external name by which the web service will be known
2. Specify the download schedule, content, and working directories
3. Create the data web service by modifying the `GenericWS` template
4. Deploy on Tomcat

1. Specify the external name by which the web service will be known: Like the proxy web service, the `web.xml` file (Figure S5) specifies the name of the web service when it is deployed. The developer is able to leave the class and package names in the current form, however for readability when dealing with multiple web services, change the names to match the name of the new web service (e.g., `EnrichWS`). The `web.xml` in Figure S5 provides an example that accesses the service via `http://host.edu/GenericWS.` Contrasting the `web.xml` in Figure S3, the `<url-pattern> /*` specifies that there is no required path to use this web service.

```
<?xml version="1.0" encoding="utf-8"?>
<!DOCTYPE web-app PUBLIC "-//Sun Microsystems, Inc.//DTD Web Application
2.3//EN" "http://java.sun.com/dtd/web-app_2_3.dtd"[]>
<web-app>
    <servlet>
       <servlet-name>GenericWS</servlet-name>
       <servlet-class>
          genericws.GenericServlet
       </servlet-class>
       <load-on-startup>1</load-on-startup>
    </servlet>

    <servlet-mapping>
       <servlet-name>GenericWS</servlet-name>
       <url-pattern>/*</url-pattern>
    </servlet-mapping>
</web-app>
```

Figure S5 – data web service `web.xml` example

2. Specify the download schedule, content, and working directories: The `settings.xml` file contains the specification of the download schedule and specifies the following items:

- The directories used by the web service (`<DownloadDir>` and `<VersionsDir>`)
- The sources and schedule for the downloads (`<File>`)
- The provenance data management (`<FileManagement>`)
- The proxy that manages communication between the data web service and external data sources (`<ProxyURL>`).

Figure S6 shows an example of `settings.xml` for a web service that specifies downloading human gene information at 3 am every Monday from NCBI.

```
<?xml version="1.0" encoding="utf-8"?>
<!DOCTYPE WebServices SYSTEM "edu.utsa.cs.webservices.dtd">
<WebServices>
    <DownloadDir>/www/webServiceData/GenericWSnew/downloads/</DownloadDir>
    <VersionsDir>/www/webServiceData/GenericWSnew/versions/</VersionsDir>
    <File>
       <HostName>ncbi</HostName>
       <Url>ftp://ftp.ncbi.nlm.nih.gov/gene/DATA/GENE_INFO/Mammalia
                /Homo_sapiens.gene_info.gz</Url>
       <Comment>updated weekly</Comment>
       <FileType>gz</FileType>
       <FileToCreate>geneInfo9606</FileToCreate>
       <Schedule>
          <Type>Weekly</Type>
          <Day>1</Day>
          <Time>3</Time>
       </Schedule>
    </File>
    <FileManagement>
       <HoursBeforeDelete>2160</HoursBeforeDelete>
    </FileManagement>
    <ProxyURL>http://localhost/ProxyWS/Proxy?url=</ProxyURL>
</WebServices>
```

Figure S6 – sample data web service `settings.xml` file

Frequency options include Monthly (`<Day>` and `<Time>`), Weekly (`<Day>` and `<Time>`), and Daily (`<Time>` only). The developer may specify comments about the file such as current build, updating policies, or other source information. This information is transmitted to the clients when they request version information. The unzipped file will be: `geneInfo9606`.

3. Create the data web service by modifying the GenericWS template: The classes that the developer must implement extend SideCache base classes and provide the primary storage and functionality of the web service. The `GenericServlet.java`, `GenericWS.java`, and `GenericRebuildableData.java` files shown in the `src` directory of Figure S4 provide a working example for the developer to expand for a data web service specification. The developer will only change one line in the servlet class to instantiate an instance of the particular web service class.

The service class (`GenericWS`) has several methods that provide the functionality specific to the new web service. The `processFiles()` method parses the downloaded files and writes any working files to the `VersionsDir`. The `createRebuildableData()` method constructs any data structures needed by the web service. The `performOperation()` method parses the incoming queries and executes the proper methods to create the results to return.

In the `RebuildableData` class, the developer defines the data required to respond to any user query. In the multi-threaded Apache environment, the `performOperation()`, `processFiles()`, and `createRebuildableData()` methods have the potential to create a situation where the user gets a mixture of old and new data. The `RebuildableService` base class methods are carefully synchronized so that the data in the `RebuildableData` class

remains consistent. This infrastructure and its synchronization are transparent to developers as they implement new web services.

4. Deploy on Tomcat: Similar to the proxy web service, the developer combines all of the subdirectories under the `GenericWS` directory in Figure S4. The `lib` subdirectory should contain all of the imported jar files, the classes subdirectory should contain the compiled classes for the newly created project, and the `ws_config` subdirectory should contain the download specifications. After creating the WAR file but prior to deployment, the developer must correctly set permissions for the working directories. Although Tomcat creates the download and versioning directories if they do not exist, the Tomcat user must own the directories that it creates files in. For this example, `/www/webServiceData/GenericWSnew/downloads/` specifies the path for file downloads. The `/www` directory is potentially used by several services, so permissions to it are left alone. The `webServiceData` directory holds the data for all of the web services we implemented. Its permissions are set using the following Linux command:

```
chown –R tomcat /www/webServiceData/
```

Although the developer is able to specify the same download and versioning directories for multiple web services, each web service should specify a unique directory within `webServiceData` to maintain readability and separation between distinct web service entities.

The actual deployment follows the same pattern as the deployment of a proxy web service. During development, it is often necessary to deploy multiple iterations of the same servlet. This is managed by both deployment options. When Tomcat detects a change in the currently deployed WAR file, it reloads the servlet.

Currently, if a user accesses the data web service when the data web service is starting up with no previously downloaded data file, the service returns an error message indicating the user

needs to try the service again later (`"Web service is not ready check back in 10 minutes"`). This gives the data web service time to download and process the files specified in the `settings.xml` file and to create the new data blob. In subsequent deployments, the previously downloaded files will still remain in the downloads directory and the web service should start after the data blob is created.

Within the `GenericWS` data web service are several examples of typical web service tasks. Also provided are examples of returning multiple types of information including html, xml, flat and compressed files. The sample service parses download files and includes several examples of handling different parameters. It also has an example of returning provenance information to the user through a parameter call and managing and accessing versioned data.
